# Heart Failure Medical Therapy: A Review for Structural/Interventional Cardiologists

**DOI:** 10.1016/j.shj.2022.100082

**Published:** 2022-10-22

**Authors:** Alexandra Pipilas, Trejeeve Martyn, JoAnn Lindenfeld

**Affiliations:** a Department of Cardiovascular Medicine, Boston University, Boston, Massachusetts, USA; b Department of Cardiovascular Medicine, Cleveland Clinic, Cleveland, Ohio, USA; c Division of Cardiovascular Medicine, Vanderbilt University Medical Center, Nashville, Tennessee, USA

**Keywords:** Cardiology, Heart failure, Medical therapy

## Abstract

Medical therapy for heart failure (HF) has expanded rapidly in the last decade contributing to improved morbidity and mortality for patients living with HF. The indicated treatments have been traditionally stratified based on left ventricular ejection fraction. The optimization of HF medical therapy is important for interventional and structural cardiologists as HF remains among the most common causes of periprocedural hospitalization and death. Additionally, optimization of medical therapy for HF prior to the utilization of device-based therapies as well as enrollment in clinical trials is crucial. This review will serve to highlight medical therapy indicated across the left ventricular ejection fraction strata.

## Background

Heart failure (HF) is a complex clinical syndrome consisting of cardinal signs and symptoms due to a structural or functional cardiac abnormality.^1^ Over the last decade, new medical and device therapies for HF have been approved, contributing to improved morbidity and mortality in HF. The medical therapies indicated for HF have traditionally been based on left ventricular ejection fraction (LVEF)—HF with reduced ejection fraction (HFrEF, ≤40%), HF with mid-range ejection fraction (HFmrEF, 41%-49%), and HF with preserved ejection fraction (HFpEF, >50%).^2^ Despite proven benefits in quality of life, mortality, and HF-related hospitalization, as well as clinical practice guidelines to support the initiation and titration of comprehensive medical therapy, the prescription rates are low when measured in national registries.^3^

Optimization of HF medical therapy has taken on increased importance among interventional and structural cardiologists given that HF remains among the most common causes of hospitalization and death following intervention.^4^^,^^5^

The optimal treatment of HF in the periprocedural setting could allow for reduction in morbidity both before and after the procedure. Contemporary device trials, such as the Cardiovascular Outcomes Assessment of the MitraClip (Abbott, Santa Clara, California) Percutaneous Therapy for Heart Failure Patients with Functional Mitral Regurgitation (COAPT) study, have required maximally tolerated guideline-directed medical therapy (GDMT) as determined by an HF physician prior to device use.^6^ A similar study, the Percutaneous Repair with the MitraClip Device for Severe Functional/Secondary Mitral Regurgitation (MITRA-FR) trial, did not require the same degree of GDMT optimization prior to enrollment, and there were significant adjustments made to medical therapy during the trial. The difference in the degree of medical optimization prior to enrollment in these two trials has often been cited as one of the reasons the MITRA-FR study did not show the same degree of beneficial effect of the intervention when compared to the COAPT cohort.^7^ If GDMT is not optimized prior to an intervention being studied in a clinical trial, differential use in a control or treatment group after the intervention could affect study outcomes. Once GDMT is optimized, it is important to ensure that the patient remains symptomatic prior to the consideration of device-based intervention both in clinical practice and when considering them for device-based trials. Given the often-complex nature of medical therapy optimization as well as patient selection for both device-based therapies and complex revascularization procedures, comprehensive care with a multidisciplinary heart team is recommended.^8^^,^^9^

This review will serve to highlight the recent advances in medical therapy and outline what currently defines optimal medical therapy across the LVEF spectrum. A summary of indicated medical therapy stratified by EF is provided in Table 1.

**Table 1:** Recommendation summary

<40%	Class of recommendation		

	1	BBs	
		Bisoprolol	Recommended to reduce morbidity and mortality
		Metoprolol	
		Carvedilol	

		Renin angiotensin system inhibition	

		ARNI	In symptomatic patients, recommended to reduce morbidity and mortality.

		ACEi	If ARNI is not feasible

		ARB	If ACEi intolerant and ARNI is not feasible

		Mineralocorticoid antagonists	

		Spironolactone	In symptomatic patients, recommended to reduce morbidity and mortality
		Eplerenone	

		SGLT-2 inhibitors	

	Dapagliflozin	In symptomatic patients, recommended to reduce hospitalization and CV mortality
	Empagliflozin	

	Diuretics	

Torsemide	In patients with fluid retention, recommended to relieve congestion and prevent worsening HF
Furosemide	
Bumex	

Hydralazine/dinitrates	

	In self-identified African American patients, with III-IV symptoms, on OMT, recommended to reduce morbidity and mortality

2a		In patients who cannot be given ACE/ARB/ARNI due to drug intolerance or renal disease

	If channel inhibitor	

Ivabradine	Recommended in patients on maximally tolerated betablocker whose HR remains >70

2b	Na/K ATPase	

Digoxin	Symptomatic despite max tolerated GDMT, can be considered to reduced hospitalizations

“Improved EF”	1		GDMT should be continued to prevent relapse of HF and LV dysfunction

41%-49%	1	Diuretics	In patients with fluid retention, recommended to relieve congestion and prevent worsening HF

	2a	SGLT-2	Can be beneficial in decreasing HF hospitalizations and CV mortality

2b	ACE/ARB/ARNI, MRA, BB	Among symptomatic patients, can be considered to reduce HF hospitalization and CV mortality

>50%	2a	SGLT-2	Can be beneficial in decreased heart failure hospitalizations and CV morality
	2b	MRA	May be considered to decrease hospitalizations
		ARNI	May be considered to decrease hospitalizations

ACC, American College of Cardiology; ACEi, angiotensin-converting enzyme inhibitor; AHA, American Heart Association; ARB, angiotensin receptor blocker; ARNI, angiotensin receptor-neprilysin inhibitor; BB, beta blocker; CV, cardiovascular; EF, ejection fraction; GDMT, guideline-directed medical therapy; HF, heart failure; HFSA, Heart Failure Society of America; HR, heart rate; LV, left ventricle; MRA, mineralocorticoid receptor antagonist; OMT, optimal medical therapy; SGLT-2, sodium-glucose cotransporter 2.

Adapted from 2022 AHA/ACC/HFSA Guideline for the management of heart failure.

As the inpatient setting is known to be a critical period to initiate and titrate GDMT,^10^ this review will also provide an overview of relevant observational and randomized data supporting the safety and efficacy of inpatient initiation of GDMT. Lastly, although there is little high-quality evidence to guide the appropriate sequence or prioritization of GDMT, we will provide a brief commentary on patient selection for pharmacotherapy in HFrEF.

## Heart Failure With Reduced Ejection Fraction

### Foundational Therapies

The foundation of medical therapy for HFrEF centers on the inhibition of the renin-angiotensin-aldosterone system (RAAS) with the use of angiotensin-converting enzyme inhibitors (ACEis), angiotensin receptor blockers (ARBs) or angiotensin receptor-neprilysin inhibitor (ARNI), mineralocorticoid receptor antagonist (MRA), the inhibition of the sympathetic nervous system with beta-blockers, and the more recent addition of sodium-glucose cotransporter 2 (SGLT-2) inhibitors. The data for the use of these 4 pillars of medical therapy will be the focus of this portion of the review. The major landmark trials supporting the use of these therapies are outlined in Table 2. Common indications, starting and goal medication doses, and contraindications for these medications are provided in Table 3.

**Table 2:** Primary results from recent major phase III clinical trials

HFrEF		Study population	Patients (n)	Follow-up, mo	Primary outcome	Event rate, %		HR (95% CI)	*p* Value
						Study drug	Control		
ACE inhibitors									
CONSENSUS	Enalapril vs. placebo	NYHA class IV	253	6	6-mo Mortality	26	44	RR 0.6	0.002
SAVE	Captopril vs. placebo	Acute MI, symptomatic HF	2231	42	All-cause mortality	20.4	24.6	0.81 (0.68-0.97)	0.02
SOLVD	Enalapril vs. placebo	EF <35%, NYHA II-IV	2569	48	All-cause mortality	35.2	39.7	0.84 (0.74-0.95)	0.003
AIRE	Ramipril vs. placebo	Acute MI, symptomatic HF	2006	15	All-cause mortality	16.7	22.4	0.73 (0.6-0.89)	0.002
ARB									
Val-HeFT	Valsartan vs. placebo	NYHA II-IV	5010	23	All-cause mortality	19.7	19.4	RR 1.02 (0.88-1.18)	0.80
					Mortality, HF hospitalization, cardiac arrest	28.8	32.1	RR 0.87 (0.77-0.97)	0.009
ARNI									
PARADIGM-HF	Sacubitril-valsartan vs. enalapril	NYHA II-III	8842	27	All-cause mortality	17.0	19.8	0.84 (0.76-0.93)	<0.001
LIFE	Sacubitril-valsartan vs. valsartan	NYHA class V	335	6	Change in NT-proBNP	--	--	1.32 (0.86-2.03)	0.20
PARADISE-MI	Sacubitril-valsartan vs. ramipril	High-risk AMI, LVEF <40% + evidence of congestion	5661	23	CV death, first hospitalization for HF, opt HF	11.9%	13.2%	0.9 (0.78-1.04)	0.17
PIONEER-HF	Sacubitril-valsartan vs. enalapril	ADHF	881	2	Change NT-proBNP	-47%	-25%	0.71 (0.63-0.81)	<0.001
Beta blockers									
CIBIS II	Bisoprolol vs. placebo	NYHA III-IV	2647	15.6	All-cause mortality	11.8	17.3	0.66 (0.54-0.81)	<0.001
					Sudden cardiac death	3.6	6.3	0.56 (0.39-0.80)	0.001
MERIT-HF	Metoprolol succinate vs. placebo	NYHA II-III	3991	12	All-cause mortality	7.2	11.0	0.66 (0.53-0.81)	<0.001
					Deaths from HF	30	58	0.51 (0.33-0.79)	0.002
COPERNICUS	Carvedilol vs. placebo	NYHA III-IV	2289	10.4	All-cause mortality	12.8	19.7	RR 0.65 (0.52-0.81)	0.00013
					CV hospitalization	14.1	19.6	0.73 (0.55-0.97)	0.04
Mineralocorticoid receptor antagonist									
RALES	Spironolactone vs. placebo	EF <35%, NYHA III-IV	1663	24	All-cause mortality	35	46	0.7 (0.6-. 82)	<0.001
					HF hospitalization	26.1	35.7	0.65 (0.54-0.77)	<0.001
EPHESUS	Eplerenone vs. placebo	AMI, HF symptoms	6642	16	All-cause mortality	14.4	16.7	0.85 (0.75-0.96)	0.009
					CV morality or hospitalization for CV event	26.7	30	0.87 (0.79-0.85)	0.002
EMPHASIS-HF	Eplerenone vs. placebo	NYHA class II	2737	21	CV death or HF hospitalization	18.3	25.9	0.63 (0.54-0.74)	0.001
SGLT-2 inhibitor									
DAPA-HF	Dapagliflozin vs. placebo	NYHA II-IV	4744	18.2	Worsening HF or CV death	16.3	21.2	0.74 (0.65-0.85)	0.001
EMPEROR-Reduced	Empagliflozin vs. placebo	NYHA II-IV	3730	16	CV death or first hospitalization for HF	19.4	24.7	0.75 (0.65-0.85)	0.001
SOLIST–WHF	Sotagliflozin vs. placebo	Admission with HF (mean EF 35%)	1222	9 mo	CV death or hospitalization for HF	70 Events/100 patient y	98 Events/100 patient y	0.67 (0.52-0.85)	0.0009
EMPULSE	Empagliflozin vs. placebo	ADHF	530	90 d	Death, number of HF events, symptom change	53.9	39.7	------	0.0054
Vasodilators									
A-HeFT	Hydralazine/isosorbide dinitrate	Self-identified African American patients, NYHA III-IV	1050	10 mo	All-cause mortality	6.2	10.2		0.02
Ivabradine									
SHIFT	Ivabradine vs. placebo	NYHA II-IV	6558	22.9	Death from HF or hospitalization	24	29	0.82 (0.75-0.9)	<0.0001
Soluble guanylate stimulator									
VICTORIA	Vericiguat vs. placebo	NYHA II-IV	5050	12	CV death or hospitalization for HF	35.5	38.5	0.90 (0.82-0.98)	0.02
Myosin activator									
GALACTIC-HF	Omecamtiv mecarbil vs. placebo	NYHA II-IV	8256	21.8	First HF event or CV death	37	39.1	0.92 (0.86-0.99)	0.03

ACE, angiotensin-converting enzyme; ADHF, acute decompensated heart failure; A-HeFT, The African-American Heart Failure Trial; AIRE, Acute Infarction Ramipril Efficacy; AMI, acute myocardial infarction; ARB, angiotensin receptor blocker; ARNI, angiotensin receptor-neprilysin inhibitor; CI, confidence interval; CIBIS II, cardiac insufficiency bisoprolol study II; COPERNICUS, carvedilol prospective randomized cumulative survival; CV, cardiovascular; DAPA-HF, Dapagliflozin and Prevention of Adverse Outcomes in Heart Failure; EF, ejection fraction; EMPEROR-Reduced, EMPagliflozin outcomE tRial in Patients With chrOnic HF With Reduced Ejection Fraction; EMPHASIS-HF, Eplerenone in Mild Patients Hospitalization and Survival Study in Heart Failure; EPHESUS, Eplerenone Post-Acute Myocardial Infarction Heart Failure Efficacy and Survival Study; GALACTIC-HF, Global Approach to Lowering Adverse Cardiac outcomes Through Improving Contractility in Heart Failure; HF, heart failure; HFrEF, HF with reduced ejection fraction; HR, hazard ratio; LVEF, left ventricular ejection fraction; MERIT-HF, metoprolol CR/XL randomised intervention trial in congestive heart failure; MI, myocardial infarction; NT-proBNP, N-terminal pro–B-type natriuretic peptide; NYHA, New York Hospital Association; PARADIGM-HF, Prospective Comparison of ARNI with ACEi to Determine Impact on Global Mortality and Morbidity in HF; PARADISE-MI, ACE inhibitor trial to DetermIne Superiority in reducing heart failure Events after Myocardial In- fraction; PIONEER-HF, Comparison of Sacubitril–Valsartan vs. Enalapril Effect on NT-proBNP in Patients Stabilized from an Acute HF Episode; SGLT-2, sodium-glucose cotransporter 2; RALES, Randomized Aldactone Evaluation Study; RR, relative risk; SAVE, Survival and Ventricular Enlargement trial; SHIFT, Systolic Heart Failure Treatment With the IF Inhibitor Ivabradine Trial; SOLIST–WHF, Effect of Sotagliflozin on Cardiovascular Events in Patients With Type 2 Diabetes Post Worsening Heart Failure; SOLVD, Studies of left ventricular dysfunction; Val-HeFT, Valsartan Heart Failure Trial; VICTORIA, Vericiguat Global Study in Subjects With Heart Failure with Reduced Ejection Fraction.

**Table 3:** Major medical therapy

Drug class	Common indications	Starting dose		Target dose	Contraindications
ACEi	EF <40%, NYHA class I-IV patients who cannot tolerate ARNI∗	Captopril	6.25 mg 3 times a day	50 mg 3 times a day	Advanced CKD History of hyperkalemia History of angioedema
		Enalapril	2.5 mg twice a day	10-20 mg twice a day	
		Lisinopril	2.5-5 mg daily	20-40 mg daily	
		Ramipril	1.25-2.5 mg daily	10 mg daily	
		Fosinopril	5-10 mg daily	40 mg daily	
ARB	EF < 40%, NYHA I-IV who cannot tolerate or in whom ACE/ARB is not feasible∗ EF >40%	Candesartan	4-8 mg daily	32 mg daily	Advanced CKD History of hyperkalemia
		Losartan	25-50 mg daily	150 mg daily	
		Valsartan	20-40 mg daily	160 mg twice a day	
ARNI	EF <40%, NYHA II-IV∗ EF >40%	Sacubitril-valsartan	24-26 mg daily	96-103 md twice a day	Advanced CKD History of hyperkalemia History of angioedema
Mineralocorticoid antagonists	EF <35%, NYHA class II-III∗ EF >40%	Spironolactone	12.5-25 mg daily	25 mg daily	History of hyperkalemia CKD: Women: Cr > 2.0 Men: Cr > 2.5
		Eplerenone	25 mg daily	50 mg daily	
Betablockers	EF <40%, NYHA I-IV∗ EF 41-49%	Bisoprolol	1.25 mg daily	10 mg daily	Low output heart failure Symptomatic bradycardia
		Carvedilol	3.125 mg twice a day	25-50 mg daily	
		Metoprolol Succinate	12.5-25 mg daily	200 mg daily	
SGLT-2	EF <40%, NYHA II-IV∗ EF >40%	Dapagliflozin	10 mg daily	10 mg daily	Patients on dialysis
		Empagliflozin	10 mg daily	10 mg daily	
If channel inhibitor	NYHA class II to II, EF <35%, on a beta blocker at maximum tolerated dose, and who are in sinus rhythm with a heart rate of >70 bpm at rest	Ivabradine	5 mg twice a day	40 mg/300 mg 3 times a day	Isosorbide: concomitant use of PDE-5 inhibitors

ACC, American College of Cardiology; ACEi, angiotensin-converting enzyme inhibitor; AHA, American Heart Association; ARB, angiotensin receptor blocker; ARNI, angiotensin receptor-neprilysin inhibitor; Cr, creatinine; EF, ejection fraction; GDMT, guideline-directed medical therapy; HFrEF, heart failure with reduced ejection fraction; NYHA, New York Hospital Association; PDE-5, Phosphodiesterase 5; SGLT-2, sodium-glucose cotransporter 2.

∗ Class I indication in the 2022 ACC/AHA HF guidelines.

#### Angiotensin-Neprilysin Inhibitors

Neprilysin is a neutral endopeptidase, and its inhibition serves to increase the concentration of natriuretic peptides such as brain natriuretic peptide (BNP), bradykinin, and substance P. Higher levels of these substances leads to increased natriuresis and systemic vasodilation.^11^ The combination of the neprilysin inhibitor, sacubitril, and valsartan has demonstrated incremental benefits over ACEi alone in morbidity and mortality in patients with HFrEF. The PARADIGM-HF study (Prospective Comparison of ARNI with ACEi to Determine Impact on Global Mortality and Morbidity in HF) found that sacubitril/valsartan, when compared to enalapril, reduced the composite endpoint of cardiovascular (CV) mortality, all-cause mortality, and HF hospitalizations in patients with chronic HFrEF.^12^ There was also an improvement in health-related quality-of-life scores with sacubitril-valsartan when compared to enalapril in the PARADIGM population.^13^

While patients in the PARADIGM-HF study were ambulatory New York Hospital Association (NYHA) class II-III, those enrolled in the PIONEER-HF (Comparison of Sacubitril–Valsartan vs. Enalapril Effect on NT-proBNP in Patients Stabilized from an Acute HF Episode) were hospitalized with acute HF. In this trial, there was no difference in the safety and tolerability of ACEi vs. ARNI with regard to acute kidney injury (AKI), symptomatic hypotension, or hyperkalemia suggesting de novo initiation of sacubitril-valsartan is reasonably safe in the hospitalized patient.^14^ Similarly, in the open-label TRANSITION trial, there was no difference in safety outcomes when ARNI was started either before or after discharge.^15^

The LIFE trial studied patients with a recent history of NYHA class IV symptoms. This trial found that the ARNI when compared to valsartan was not superior with respect to lowering N-terminal pro–B-type natriuretic peptide (NT-proBNP) levels at 24 weeks. However, there was no difference in safety outcomes suggesting tolerability of ARNI even in some patients with more advanced HF.^16^ Additional benefits of ARNI over other ACEi or ARB include a reduction in the decline in estimated glomerular filtration rate (eGFR),^17^ reduction in rates of hyperkalemia,^18^ and the possibility of a reduction in loop diuretic dose.^18^

Multiple recent studies have examined the use of ARNI in both acutely hospitalized and ambulatory patients, many of whom had not been on an ACEi or ARB previously. Initiation of ARNI in these patients appears to be safe and effective.^14^^,^^15^^,^^19^^,^^20^ Given the safety, tolerability, and efficacy of ARNI in the acute setting and the benefit demonstrated in chronic HFrEF patients, de novo initiation of ARNI is now a class I indication.^8^ The 2022 American College of Cardiology (ACC)/American Heart Association (AHA) guidelines now recommend the use of ARNI in all symptomatic patients with LVEF less than 40% to reduce morbidity and mortality, regardless of prior ACE/ARB use. ACEi should be used when ARNI is not feasible or not tolerated, and patients who have previously tolerated ACEi or ARB should be transitioned to ARNI.^8^

#### ACE Inhibitors/Angiotensin Receptor Blockers

Dysregulation of the RAAS is central to the pathophysiology and progression of the HF syndrome. The RAAS is activated by renal hypoperfusion and sympathetic activation. This activation leads to vasoconstriction, water and salt retention, and further sympathetic activation all exacerbating the HF syndrome.^21^ Numerous studies have shown that inhibition of the RAAS system with ACEi and ARB improves morbidity and mortality in patients with a reduced EF. The reduction in mortality is on the order of 20% to 30%.^20^^,^^22^^,^^23^ Because of this, the use of ACEi/ARB in patients who cannot take an ARNI carries a class I indication in HFrEF.^22^, ^23^, ^24^ A meta-analysis^25^ of several large clinical trials showed that high doses of ACEi and ARBs reduce all-cause mortality when compared with a low dose with no significant difference in the risk of HF hospitalization or several safety endpoints including hypotension or renal failure. High-dose ACEi or ARB was associated with a 2-fold higher incidence of hyperkalemia, so caution must be taken in patients with such history.

The initiation of ACEi or ARBs in patients hospitalized with acute HF has been shown to be safe and associated with improved outcomes at 1 ​year.^26^ There are often questions regarding the use of ACE/ARB prior to invasive procedures. While there are pilot data to suggest the holding of ACEi/ARB prior to cardiac catheterization may prevent AKI,^27^ the data are inconclusive and do not conclusively support the holding or initiation of ACEi/ARB therapy.^28^

There is significant observational evidence to support a potential role for RAAS blockade therapy in severe aortic stenosis given its association with modulating left ventricular remodeling, reducing LV hypertrophy and fibrosis, and known clinical benefit in the HF population.^23^^,^^29^^,^^30^ The Ramipril in Aortic Stenosis (RIAS) trial is one prospective, randomized, double-blind, placebo-controlled trial that evaluated the ACEi in patients with asymptomatic moderate or severe aortic stenosis.^31^ One hundred patients were randomized to ramipril or placebo for 1 year. When compared to placebo, ACEi led to a significant reduction in LV mass.

#### Beta Blockers

Beta blockers (BBs) reduce the deleterious effects of excess catecholamine stimulation in patients with HFrEF.^32^ The use of an evidence-based BB (metoprolol succinate, carvedilol, or bisoprolol) has been shown to reduce all-cause death, CV death, sudden cardiac death, and HF hospitalizations in patients with HFrEF.^33^, ^34^, ^35^, ^36^ The benefits of betablockers are also seen in patients with more-advanced HF^37^ and elderly patients.^38^ The current recommendation is that all patients without a clear contraindication be placed on BBs as soon as the diagnosis of HFrEF is established.^8^ The initiation of betablockers in the acute setting should be done cautiously and once the patient is decongested and stabilized. BBs should be used with caution in patients who are known to have low cardiac output,^2^ and these patients may be good candidates for referral to a cardiologist with experience in advanced HF.

#### Mineralocorticoid Antagonists

MRAs work to block the effects of upregulated aldosterone, produce a weak diuretic effect, and lower blood pressure in patients with HFrEF.^39^ Three major clinical trials demonstrated that MRAs improved progressive HF death and sudden cardiac death by 15% to 30%, decreased hospitalizations by 15% to 40%, and improved quality of life in patients with symptomatic HF.^40^, ^41^, ^42^ MRA should be added for all patients with a reduced EF and at least NYHA class II symptoms.^8^ MRA has also been shown to be beneficial for patients with a reduced EF in the postmyocardial infarction setting.^43^ MRA should be discontinued in patients in whom serum potassium cannot be maintained less than 5.5 mEq/L and should not be used in women with serum creatinine above 2.0 mg/dL and in men with creatinine above 2.5 mg/dL^8^ because of the excess risk of hyperkalemia.

#### Sodium-Glucose Cotransporter 2 Inhibition

The mechanism by which the SGLT-2 inhibitors (SGLT-2i) lead to improved CV outcomes is incompletely understood. Multiple mechanisms have been proposed including diuretic and antihypertensive effects, improved CV energetics, induction of autophagy, and increased hemoglobin.^44^ The Dapagliflozin and Prevention of Adverse Outcomes in Heart Failure (DAPA-HF) trial found a 26% reduction in the composite endpoint of worsening HF or CV death in symptomatic ambulatory HF patients already on GDMT with ACEi/ARB/ARNI, MRA, and BB. The benefits were seen regardless of diabetes status. In the EMPEROR-Reduced (EMPagliflozin outcomE tRial in Patients With chrOnic HF With Reduced Ejection Fraction) trial, the SGLT-2i empagliflozin reduced the primary composite endpoint of CV death or HF hospitalizations by 25% in a similar patient population, driven primarily by a reduction in HF hospitalizations. There was also an improvement in HF-related quality-of-life scores.^45^

Clinical trial data would suggest that SGLT-2i therapy is an attractive therapy across a broad range of blood pressures and renal function. The EMPEROR-Reduced trial included patients with an eGFR >20 while DAPA-HF included those with an eGFR >30. In a substudy of the DAPA-HF population, SGLT-2i therapy lowered systolic blood pressure by 2.5 mmHg, suggesting a very modest impact on blood pressure.^46^ Additionally, there is a rapid time to benefit with initiation of SGLT-2i which should compel broad and rapid implementation of this therapy in eligible patients.^47^^,^^48^

Given the established benefit in ambulatory patients with HF, the SOLOIST (Effect of Sotagliflozin on Cardiovascular Events in Patients With Type II Diabetes And Worsening HF) and EMPLUSE trials evaluated the initiation in patients with recently stabilized acute HF and found a similar significant clinical benefit. There was a favorable safety profile and a reduction in death and hospitalization for HF. Notably the clinical benefit was evident within 90 days of randomization supporting the rapid benefit and safety of SGLT-2 in these patients.^47^^,^^48^

An additional benefit of the SGLT-2i that was noted in early trials was a reduction in the rate of decline in GFR. The DAPA-CKD trial found that when compared to placebo, dapagliflozin reduced the risk of a composite of a sustained decline in the estimated GFR of at least 50%, end-stage kidney disease, or death from renal or CV in a cohort of patients with CKD with proteinuria and an eGFR at baseline between 25 and 75 mL/min/1.73 m^2^.^49^

In the 2022 HF guidelines, an SGLT-2i is recommended for all patients with chronic symptomatic HFrEF, regardless of diabetes status, to reduce hospitalization for HF and CV mortality.^8^

The use of SGLT-2i in the posttranscatheter aortic valve replacement setting is the subject of an ongoing clinical trial.^50^

### Timing of Medication Initiation and Uptitration HFrEF

The optimal timing and sequencing of HF medications has not been studied systematically and has been an active area of debate among HF experts.^51^

Often medications are added in a stepwise fashion beginning with those that were studied first with the newest drug classes being added after titration of prior drugs to maximally tolerated doses. This is despite evidence of the early clinical benefit of several of the newer drug classes and significant aggregate clinical benefit of all 4 drug classes given together.^52^ Some experts have even proposed a method to “grade” or quantify the overall quality and dosing of evidence-based therapy through a GDMT scoring system.^53^ Some have proposed a personalized approach with uptitration of medications based on certain presenting phenotypes centered around a patient’s baseline heart rate, blood pressure, and renal function.^54^

We suggest the prioritization of RAAS inhibition for patients with baseline elevated blood pressures, type II diabetes, or proteinuric kidney disease. They should be avoided in patients with AKI. More aggressive uptitration of BBs or the addition of ivabradine may be beneficial in patients with baseline elevated heart rates, concomitant coronary artery disease (CAD) or atrial arrhythmias. BB should be titrated to the maximally tolerated dose. Ivabradine may be considered if heat rate is ≥70 after maximally tolerated doses of BBs are achieved. MRA should be considered for patients who struggle with hypokalemia or resistant hypertension but avoided in those with a history of hyperkalemia or significant renal dysfunction.^8^ SGLT-2i are very well tolerated with only very modest effect on blood pressure even in recently stabilized patients. Because of this, we favor the early addition of SGLT-2i.

As the HF syndrome progresses, intolerance to GDMT including dose reductions in therapy along with elevations in natriuretic peptides, recurrent hospitalizations, and/or worsening LV function are danger signals that should prompt referral to an advanced HF specialist for consideration of advanced therapies.^8^ In the acute HF setting, determination of the optimal medical regimen especially for patients with end-organ dysfunction is often complex and beyond the scope of this review. This is detailed extensively in guidelines by the European Society of Cardiology.^55^

In patients with chronic HF, BNP or NT-proBNP measurements can be used for risk stratification. Elevated levels may help to identify patients at higher risk who may benefit from additional optimization. NT-proBNP cutoffs have been used in multiple recent large clinical trials to establish eligibility criteria.^14^^,^^45^^,^^56^ In the future, NT-proBNP cutoffs may be used to determine drug and device eligibility.

### Additional Therapies for HFrEF

If a patient has been optimized on the maximally tolerated doses of the 4 pillars of GDMT described above and remains symptomatic, there are some additional medical therapies that can be considered. In this setting, we suggest referral to an advanced HF cardiologist.

#### Omecamtiv Mecarbil

Omecamtiv mecarbil, a selective cardiac myosin activator, is part of a new class of drugs known as myotropes which act directly on the cardiac sarcomere to directly improve myocardial function.^57^ It has been shown to increase the left ventricular systolic ejection time, which in prior studies has been linked to improved CV outcomes and increase in stroke volume after 20 weeks.^58^ The subsequent Global Approach to Lowering Adverse Cardiac outcomes Through Improving Contractility in Heart Failure study demonstrated a small (8%) improvement in the composite endpoint of CV death or first HF event, largely driven by reduction in HF events.^57^ Subsequent retrospective analyses have suggested the largest benefit in patients with very low EFs.^59^ The Acute Treatment With Omecamtiv Mecarbil to Increase Contractility in Acute Heart Failure study, although it did not show improved outcomes, did demonstrate omecamtiv’s tolerability in the acute HF setting.^60^

#### Vericiguat

Vericiguat is a soluble guanylate cyclase inhibitor which, in concert with NO, acts to increase the level of cyclic guanosine monophosphate (cGMP) leading to smooth muscle relaxation and vasodilation.^56^ In the Vericiguat Global Study in Subjects With Heart Failure with Reduced Ejection Fraction (VICTORIA) study, vericiguat demonstrated a lower incidence of the primary outcome of death from CV cause or hospitalization for HF than placebo in patients with reduced EF who remained symptomatic on maximally tolerated GDMT. The primary endpoint was driven by a reduction in HF hospitalization. Vericiguat did not significantly reduce natriuretic peptides at 12 weeks but was well tolerated with a limited side effect profile.^61^

#### Ivabradine

Ivabradine, an I_f_ inhibitor, works to slow the heart rate by working primarily at the sinus node. In the Systolic Heart Failure Treatment With the IF Inhibitor Ivabradine Trial (SHIFT trial) ivabradine reduced the combined endpoint of CV mortality and HF hospitalization in symptomatic HF patients already on GDMT with ACE/ARB, BB, or MRA who had a baseline heart rate greater than 70 bpm.^62^

#### Isosorbide Dinitrate and Hydralazine

The combination of hydralazine and isosorbide dinitrate was shown to reduce mortality and hospitalization of HF in self-identified African-American patients with symptomatic HF who were already on GDMT with ACE/ARB, BB, and MRA in the small African-American Heart Failure Trial (A-HeFT) trial.^63^ The results have not been replicated in a wider population. The addition of this drug combination should be considered in self-identified African-American patients who remain symptomatic despite maximally tolerated therapy or in patients who are unable to tolerate afterload reduction with an ACE/ARB/ARNI due to renal dysfunction.^8^

#### Digoxin

The use of digoxin can be considered for patients with HFrEF in normal sinus rhythm who remain symptomatic despite treatment with other maximally tolerated GDMT. The DIG trial showed a reduction in overall and HF-related hospitalizations but did not reduce mortality. Notably, this was without background betablocker therapy.^64^ A prespecified analysis of the Digitalis Investigation Group trial suggested substantial benefit in those with NYHA class II and IV and/or with an LVEF <25%.^65^ The addition of digoxin can be considered in patients who remain symptomatic despite other maximally tolerated GDMT to reduce HF-related hospitalizations.

## Heart Failure With Midrange Ejection Fraction

HFmrEF first appeared as a distinct subdivision of HF in the 2016 European Society of Cardiology HF guidelines. The patient phenotype and response to medical therapy most closely resemble those of patients with HFrEF. However, there is some thought that it may represent a transition phenotype between HFpEF and HFrEF.^66^ Dedicated randomized controlled trials of HF therapies in this group are needed.^2^ Outside of diuretics for symptom control, there are no medications that have been granted a class I indication for patients with HFmrEF likely due to lack of significant data.^8^ Much of the data to support the use of other GDMTs in this population are taken from extrapolation of HFrEF data, exploratory analyses, and from small portions of patients included in larger trials.

#### Angiotensin-Neprilysin Inhibitors

There have been no trials evaluating the use of ARNI in patients with HFmrEF. Similar to other types of RAAS blockade, the use has a class IIb recommendation in the most recent guideline.^8^ The data for this recommendation come from a secondary analysis of the PARADIGM-HF and Prospective Comparison of ARNI with ARB Global Outcomes in HF with Preserved Ejection Fraction (PARAGON-HF) studies. In PARAGON-HF, which did not meet its original primary endpoint, there was a significant EF-treatment interaction noted. In this analysis, sacubitril/valsartan when compared to valsartan lead to a significant reduction in the primary composite endpoint of CV death and total HF hospitalizations by 22% for patients with an EF between the 45% and 57%.^67^ In combined patient data from PARAGON-HF and PARADIGM-HF, a similar treatment effect when stratified by EF was noted with patients with an EF below 57% deriving the most benefit.^68^

#### ACE Inhibitors/Angiotensin Receptor Blockers

There are no completed trials evaluating the use of ACEi or ARB in patients with HFmrEF. The use in this population has a IIb recommendation in the most recent guidelines based on a secondary analysis of the candesartan in heart failure assessment of reduction in mortality and morbidity (CHARM-Preserved trial) which revealed a reduction in the number of HF hospitalizations with candesartan when compared to placebo.^8^

#### Mineralocorticoid Antagonist

There have been no dedicated randomized controlled trials for spironolactone in patients with HFmrEF. However, the use of spironolactone has a IIb recommendation based on a secondary analysis of the Treatment of Preserved Cardiac Function Heart Failure With an Aldosterone Antagonist (TOPCAT) trial which showed fewer hospitalizations for HF in patients with an LVEF 45% to 55%.^69^

#### Beta Blockers

There have been no dedicated randomized controlled trials evaluating the use of betablockers in patients with HFmrEF. The use of BB has a class IIb recommendation based on a meta-analysis of 11 HF trials by the BB meta-HF group which showed, in a subgroup of 575 patients with LVEF 40% to 49% in sinus rhythm, BBs reduced the primary outcome of all-cause and CV mortality.^70^

#### Sodium-Glucose Cotransporter 2 Inhibitors

There have been no trials specifically aimed at evaluating the use of SGLT-2i in patients with HFmrEF although one is planned.^29^ Given the benefit seen in EMPEROR-Preserved^68^ and EMPEROR-Reduced trials,^45^ a benefit for patients with an EF of 41% to 49% is established. In the most recent guidelines, the use of SGLT-2i for this group was given a 2a recommendation to reduce HF hospitalizations and CV mortality.

## Heart Failure With Improved Ejection Fraction

HF with an improved EF, defined as a previous LVEF <40% with subsequent evidence of improvement and LVEF >40%,^8^ now appears as an additional subcategory in the guidelines. Functionally it can be viewed as a continuation of the HFrEF phenotype. Due to the risk of relapse of systolic dysfunction with cessation of previously prescribed GDMT,^71^ the AHA/ACC guidelines recommend continuing GDMT in these patients even if they become asymptomatic.^8^

## Heart Failure With Preserved Ejection Fraction

The diagnosis of HFpEF is defined as the clinical syndrome of HF with an LVEF >50%. HFpEF represents a diverse population made up of multiple distinct phenotypes with a different underlying pathophysiology, the identification of which is paramount given some phenotypes require specific therapies.^72^ Many of the previous large randomized controlled trials for HFpEF have had negative results, resulting in a lack of effective treatments to improve both morbidity and mortality in this high-risk patient population. The results are summarized in Table 4. As the identification of specific phenotypes of clinical HFpEF improves, we may be able to better define the effects of certain treatments.

**Table 4:** Primary results from recent major phase III clinical trials

HFpEF		Study population	Patients (n)	Follow-up, mo	Outcome	Event rate, %		HR (95% CI)	*p* Value
						Study drug	Control		
ARNI									
PARAGON-HF^61^	Sacubitril-valsartan vs. valsartan	NYHA class II-III >45%	4822	35	HF hospitalization and CV mortality	894	1009	0.87 (0.75-1.01)	0.059
SGLT-2 inhibitors									
EMPEROR-Preserved^70^	Empagliflozin vs. placebo	NYHA II-IV, LVEF >40%	5988	26.2	CV death or hospitalization for HF	13.8	17.1	0.79 (0.69-0.90)	<0.0001
MRA									
TOPCAT^63^	Spironolactone vs. placebo	Symptomatic HF, LVEF >45%	3445	3.3 y	CV death, cardiac arrest or hospitalization for HF	18.6	20.4	0.89 (0.77-1.04)	0.14

ARNI, angiotensin receptor-neprilysin inhibitor; CI, confidence interval; CV, cardiovascular; EMPEROR-Preserved, EMPagliflozin outcomE tRial in Patients With chrOnic HF with preserved ejection fraction; HF, heart failure; HFpEF, HF with preserved ejection fraction; HR, hazard ratio; LVEF, left ventricular ejection fraction; MRA, mineralocorticoid receptor antagonist; NYHA, New York Hospital Association; PARAGON-HF, Prospective Comparison of ARNI with ARB Global Outcomes in HF with Preserved Ejection Fraction; SGLT-2, sodium-glucose cotransporter 2; TOPCAT, Treatment of Preserved Cardiac Function Heart Failure With an Aldosterone Antagonist.

#### Angiotensin-Neprilysin Inhibitors

The PARAGON-HF study, which compared the sacubitril/valsartan to valsartan in patients with an LVEF >45%, did not meet its primary endpoint although, as described above, there was a significant EF-by-treatment interaction noted with a benefit for patients with an EF of 45% to 57% with an extension to high EF in female patients.^67^ Given this, the ACC/AHA guidelines note that the use of ARNI can be considered in these patients to reduce hospitalizations.^8^

#### ACE Inhibitors/Angiotensin Receptor Blockers

The routine use of ACEi or ARBs in patients with HFpEF has not been proven beneficial in any large randomized controlled trials performed to date.^29^^,^^73^^,^^74^ However, many patients with HFpEF often have comorbid hypertension and kidney disease, which have strong indications for RAAS inhibition therapy. Retrospective evaluations of HFpEF trials have suggested a benefit of ARBs providing the support for a 2b recommendation to reduce HF hospitalizations in this group.

#### Beta Blocker

The routine use of BBs for patients with HFpEF is not recommended as there have been no large trials demonstrating benefit. The withdrawal of betablocker therapy in one recent trial for patients with HFpEF and chronotropic incompetence led to an improvement in peak VO2, quality of life scores, and left ventricular end-diastolic pressure.^75^ The use of the BB therapy in HFpEF requires additional research.

#### Mineralocorticoid Antagonist

The use of spironolactone in patients with HFpEF is based largely on retrospective evaluation of the TOPCAT trial which evaluated spironolactone vs. placebo in patients with an LVEF >45%. The composite primary endpoint of death from CV cause, aborted cardiac arrest, or hospitalization for HF was numerically lower in the spironolactone group but did not meet statistical significance. This difference was largely driven by a numerically lower rate of hospitalization for HF as rates of death and cardiac arrest were similar between groups. In a post hoc analysis focusing only on patients enrolled in the Americas, spironolactone did reduce the risk of the primary outcome. Notably, when compared to the placebo, the rates of hyperkalemia and increased creatine were higher in the spironolactone group.^69^ The use of spironolactone for patients with HFpEF has a 2b recommendation in the latest guidelines to reduce HF hospitalizations.^8^

#### Sodium-Glucose Cotransporter 2 Inhibitors

The SGLT-2i are the first class of drugs to demonstrate benefit in patients with HFpEF. In the EMPEROR-Preserved trial, treatment with empagliflozin lead to a 21% relative risk reduction in the composite endpoint of hospitalization for HF or CV death. This was largely driven by a reduction in HF hospitalizations. Similar to the benefit seen in patients with HFrEF, empagliflozin also lead to a lower total number of hospitalizations and a longer time to first hospitalization for HF. This effect was seen regardless of diabetes status and across LVEF strata.^76^ A sub-analysis examined the effect of empagliflozin in MRA users and nonusers in the EMPEROR-Preserved patient cohort and found empagliflozin to be beneficial regardless of background MRA use although the effect was more pronounced in MRA nonusers. The SGLT-2i also decreased the rates of hyperkalemia, regardless of MRA use.^77^

The more recent EMPULSE trial showed that among patients with acute decompensated HF, empagliflozin was associated with significant clinical benefit at 90 days, when compared to placebo. This trial demonstrated both the early clinical benefit of SGLT-2i in patients with HFpEF as well as safety and tolerability when started in the inpatient setting.^47^ SGLT-2i now have a 2a recommendation for patients with HFpEF to reduce hospitalizations and CV mortality.^8^

## Conclusions/Future Directions

HF is a complex clinical syndrome and remains a large source of morbidity and mortality globally. Familiarity and competency of non-HF clinicians with core medical therapy are crucial to ensure patients who are evaluated for invasive procedures remain symptomatic despite maximally tolerated GDMT. The identification of patients who would benefit from additional optimization of medical therapy prior to device-based intervention or prior to enrollment in clinical trials has the potential to result in better procedural outcomes and a reduction in HF hospitalization and death. Further study of the optimal use and sequencing of evidence-based therapy in sub-populations including patients with structural heart disease is needed.

Implementation of core GDMT by non-HF cardiologists in the structural intervention realm could have significant benefit to patient outcomes.^78^

## Funding

Dr Martyn receives funding support from the 10.13039/100007311Cleveland Clinic Razavi Fellowship for Clinical Research.

## Disclosure statement

Dr Martyn serves as an advisor to Recora Health and receives grant support from Ionis Therapeutics unrelated to this work. Dr Lindenfeld receives grant funding from AstraZeneca, Volumetrix, and Novo Nordisk and consulting fees from AstraZeneca, Abbott, Alleviant, Bayer, Boehringer Ingelheim, Boston Scientific, CVRx, Edwards Lifesciences, Merck, Medtronic, and VWave. Dr Pipilas has no conflicts to declare.
